# Changes in Hematological and Hemorheological Parameters Following Mild COVID-19: A 4-Month Follow-Up Study

**DOI:** 10.3390/hematolrep15040057

**Published:** 2023-10-10

**Authors:** Janina Bros, Lars Ibershoff, Emily Zollmann, Jonas Zacher, Fabian Tomschi, Hans-Georg Predel, Wilhelm Bloch, Marijke Grau

**Affiliations:** 1 Institute of Cardiovascular Research and Sports Medicine, Molecular and Cellular Sports Medicine, German Sport University Cologne, 50933 Cologne, Germany; 2 Department of Preventive and Rehabilitative Sports and Performance Medicine, Institute of Cardiovascular Research and Sports Medicine, German Sport University Cologne, 50933 Cologne, Germany; 3 Department of Sports Medicine, University of Wuppertal, 42119 Wuppertal, Germany

**Keywords:** red blood cells, COVID-19, red blood cell deformability, red blood cell aggregation, hemoglobin

## Abstract

Background: Coronavirus Disease 2019 (COVID-19) was described to affect red blood cells (RBC) in both severe and mild disease courses. The aim of this study was to investigate whether hematological and hemorheological changes that were previously described for COVID-19 patients after the acute infection state are still prominent after another 4 months to assess potential long-term effects. Methods: Hematological and RBC rheological parameters, including deformability and aggregation, were measured 41 days after infection in COVID-19 patients and non-COVID control (T0) and 4 months later in COVID-19 patients (T1). Results: The data confirm alterations in hematological parameters, mainly related to cell volume and hemoglobin concentration, but also reduced deformability and increased aggregation at T0 compared to control. While RBC deformability seems to have recovered, hemoglobin-related parameters and RBC aggregation were still impaired at T1. The changes were thus more pronounced in male COVID-19 patients. Conclusion: COVID-19-related changes of the RBC partly consist of several months and might be related to persistent symptoms reported by many COVID-19 patients.

## 1. Introduction

Since the beginning of 2020, the Coronavirus Disease 2019 (COVID-19) pandemic, induced by the severe acute respiratory syndrome coronavirus 2 (SARS-CoV-2), has been affecting the entire global population. During this pandemic, the number of COVID-19 cases increased rapidly because the virus is highly infectious and very easily transmitted [1]. Typical symptoms are breathlessness, cough, fever, ageusia, and loss of smell; however, there might also be cases with asymptomatic or mild disease courses. Reduced fitness is reported by many patients [2,3], which might be related to impaired oxygen transport and/or release by red blood cells (RBC) [4]. The SARS-CoV-2 virus has been demonstrated to invade erythroid precursors and progenitors [4] which can lead to hematopoietic stress, resulting in RBC morphological abnormalities, an inability to respond to cues from the environment, and a premature exit from the bone marrow [4]. Indeed, an altered hematological profile in COVID-19 patients has been observed in the acute phase of the disease, including lower RBC count, mean cellular hemoglobin concentration (MCHC), hemoglobin concentration (hb), and hematocrit (hct) [5]. Other findings also indicated reduced mean cellular volume (MCV) and mean cellular hemoglobin (MCH) [6]. Additionally, an increased red blood cell distribution width (RDW) has been described [4,7]. These alterations are associated with functional changes of the RBC. Many patients exhibit cold extremities and weak peripheral pulses, which might be an indicator of microcirculatory dysfunction [8]. Recent investigations revealed impaired rheological characteristics of COVID-19 RBC, including lower deformability, increased blood viscosity and RBC aggregation [9,10]. This might be related to membrane lipid remodeling and structural protein damages, affecting the cytoskeleton, which is crucial for the ability of the RBC to deform [11]. An impaired deformability is discussed to decrease the functional capacity of RBC for oxygen transport and/or release, resulting in tissue hypoxia [12]. These phenomena have been related to damages of the beta-chain of the hemoglobin or a rising formation of methemoglobin, which might increase the affinity for oxygen of the undamaged hemoglobin [4,13]. 

Alterations of the RBC have been described not only for severe infections [5], but also for mild disease courses [6]. Moreover, there are recurrent reports of COVID-19 symptoms persisting beyond the acute phase, sometimes for months after a severe infection, leading to the Long-COVID disease [14]. Back et al. (2022) documented losses of lung function and cardiorespiratory capacity one month after an infection with only mild symptoms [15]. This raises the question of whether the alterations of the RBC system persist in the long term, even in formerly mild disease courses.

Thus, the aim of this study was to investigate whether the hematological and hemorheological changes that were previously described are still prominent after a 4-month follow-up test in order to further understand the long-term effects of a mild SARS-CoV-2 infection on the RBC system.

## 2. Materials and Methods

### 2.1. Study Participants and Sample Processing

A total of *n* = 22 participants (*n* = 15 male, *n* = 7 females) were tested after SARS-CoV-2 infection. Samples were taken between October 2020 and May 2021, and the prevalent SARS-CoV-2 variants during this period were alpha, beta and delta. The infection was verified by respective PCR and antibody tests. A total of 18 participants showed mild symptoms; 4 remained asymptomatic. None of the participants were hospitalized. Moreover, no other diseases were documented. None of the participants had been vaccinated against SARS-CoV-2 at the time of this study. Age of the subjects is presented for the whole study population as well as for the sub-cohorts “COVID-19 male” and “COVID-19 female”. Age (range): 23.9 ± 4.6 years (15 to 31 years); COVID-19 male: 24 ± 5.1 years (15–31); COVID-19 female: 23.3 ± 3.7 years (20–31). All participants showed normal BMI values. They were examined 41 days (median) after their SARS-CoV-2 infection (T0) and were therefore no longer in the acute stage of disease progression at this time. A second examination was performed four months after the first examination (T1).

A NO-COVID control group, with *n* = 42 (*n* = 30 male, *n* = 12 females) participants, was tested once (T0) to assess to what extent the study parameters of the observation group had changed related to SARS-CoV-2 infection. The control group also showed normal BMI values. Age range of the control group was 23.7 ± 5.5 years (15 to 36 years); Control male: 24.1 ± 5.6 years (17–37); Control female: 23.8 ± 6.4 years (16–33). Persons of the NO-COVID group were selected from the cohort of regular examined persons without current or prior COVID-19-disease who conducted their mandatory yearly medical assessment at our center. The persons were matched in characteristics to the COVID group and had, at the time of testing, never had (a) typical COVID symptoms, (b) a positive COVID test, and (c) no specific antibodies in the blood sample.

Sample size calculation was performed with a power of 0.8 and α = 0.05 and revealed that *n* = 20 subjects per group were necessary for proper analysis. The number of examined subjects within this study is thus higher than recommended; however, we aimed to include all available participants to increase the statistical power.

Venous blood samples were collected at rest from the vena mediana cubiti into EDTA vacutainer (Becton Dickinson GmbH, Heidelberg, Germany) and immediately processed. Unless stated otherwise, the described parameters were analyzed for all tested participants.

### 2.2. Sample Analysis

#### 2.2.1. Basal Blood Parameters

A complete blood count was performed using the hematology analyzer Sysmex Digitana KX-21N (Sysmex Digitana AG, Horgen, Switzerland). The presented parameters include RBC count, hemoglobin concentration (hb), hematocrit (hct), mean corpuscular volume (MCV), mean corpuscular hemoglobin (MCH), mean corpuscular hemoglobin concentration (MCHC), and RBC distribution width (RDW).

#### 2.2.2. RBC Hemorheological Parameters

##### RBC Deformability

Deformability of the RBC was measured by ektacytometry using the LORRCA MaxSis (RR Mechatronics, Zwaag, The Netherlands) after dilution of 10 × 10^7^ RBC into polyvinylpyrrolidone (PVP) solution (29 cP at 37 °C, RR Mechatronics). The main principle has already been described in detail [16,17]. Briefly, samples were sheared in a Couette system, and nine consecutive shear stresses were applied to the samples. The diffraction pattern of a laser beam passing through the samples was analyzed by the LORRCA software for each applied shear stress, resulting in a corresponding elongation index (EI). The LORRCA software then computed the maximum elongation index (EImax), which is the maximum deformability at infinite shear stress, and SS1/2, which represents the shear stress at one-half EImax. The SS1/2:EImax ratio was then calculated from the two parameters. Thus, higher values indicate lower RBC deformability [16,17,18].

##### RBC Deformability under Osmotic Gradient

The LORRCA MaxSis also performed osmotic gradient ektacytometry (osmoscan), measuring the deformation of RBC shape at a defined shear stress and constant temperature (37 °C) under various osmotic conditions [16]. Approximately 10^9^ RBC were mixed with 5 mL of PVP and placed into the Couette system. The LORRCA software provided the following parameters as a result of the procedure: Omin represents the osmolality at which RBC deformability reaches its minimum in the hypotonic environment. EImax, the maximum deformability near the isotonic osmolality, and Ohyper reflects the hyperosmotic osmolality corresponding to 50% EImax.

##### RBC Aggregation

After the hct of the samples was adjusted to 40% using autologous plasma, aggregation of the RBC was measured at 37 °C by syllectometry using the LORRCA MaxSis. Prior to the measurement, all samples were completely oxygenated for 15 min with a Roller Mixer (Karl Hecht KG, Sondheim vor der Rhön, Germany). The oxygenated samples were transmitted to the Couette system, and changes in backscattered light were recorded over 120 s with two photodiodes and presented as a graph (syllectogram) to compute an Aggregation-Index (AI%). An iteration procedure was performed to calculate dIsc min, and afterwards, the threshold shear rate balancing RBC aggregation and disaggregation was received. This parameter defines the minimum change in backscatter intensity during the iteration process and represents the minimum shear rate where RBC aggregates start to disaggregate (y at dIsc min (s^−1^)) [19].

### 2.3. Statistics

Statistical analyses of the data were performed using GraphPadPrism software 9.3.1 (GraphPad Software, Boston, MA, USA). Data were analyzed for normal distribution using Shapiro–Wilk Test. A one-way ANOVA with the Tukey multi comparison test or Kruskal–Wallis with Dunn’s correction for multiple comparisons was performed to identify effects between Control and COVID-19 during follow-up. Statistical differences were considered significant at values of *p* < 0.05. Box-Whisker-Plot were prepared for graphical representation of the data. Otherwise, data are presented as mean ± SD. Pearson Correlation analysis was performed to reveal interrelations between SS1/2:EImax ratio and MCV.

## 3. Results

### 3.1. Blood Parameters

The blood values of the Total sample size are presented in Table 1. Statistical analysis revealed significantly lower MCV values in the COVID-19 group at T0 (*p* < 0.01) and T1 (*p* < 0.05), respectively, than in Controls. However, MCV values significantly increased at T1 compared to T0 in COVID-19 (*p* < 0.01). Further, compared to the control group, values for hb (*p* < 0.05), MCH (*p* < 0.01), and MCHC (*p* < 0.05) were significantly lower at T1 in COVID-19 subjects.

Comparing T0 and T1 in COVID-19 subjects, values for hb (*p* < 0.05), MCH (*p* < 0.01), and MCHC (*p* < 0.01) were significantly lower at T1. RBC count and RDW were not different between the groups.

Table 2 shows the blood values of the male cohort. The separation by sex revealed a similar pattern for the male group as for the total sample size. MCV was significantly lower in male COVID-19 at T0 than in the male control group (*p* < 0.01). Hb (*p* < 0.01), MCH (*p* < 0.001), and MCHC (*p* < 0.01) were significantly lower in male COVID-19 at T1 compared to the male control group. The comparison between male COVID-19 T0 and male COVID-19 T1 revealed significantly lower values for hb (*p* < 0.05), MCH (*p* < 0.05), and MCHC (*p* < 0.05) at T1 and higher values for MCV at T1 (*p* < 0.05). Again, RBC count and RDW were not different between the groups.

The blood parameters of the female cohort are presented in Table 3. Statistical analysis revealed significantly higher MCV values in female COVID-19 at T1 than at T0 (*p* < 0.05). The remaining parameters were comparable between the groups and time points.

### 3.2. Red Blood Cell Deformability

**Figure 1:**
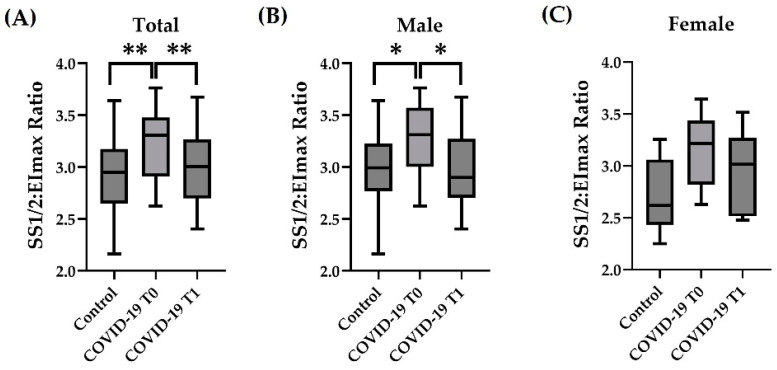
Influence of COVID-19 on RBC deformability. SS1/2:EImax Ratio of the (**A**) total sample and the (**B**) male cohort was higher in COVID-19 at T0. Values decreased from T0 to T1. (**C**) SS1/2:EImax in females showed no difference between the groups or time points. Sample size: (**A**) *n* = 41 control, *n* = 22 COVID-19; (**B**) *n* = 30 control, *n* = 15 COVID-19; and (**C**) *n* = 11 control, *n* = 7 COVID-19. * *p* < 0.05; ** *p* < 0.01.

RBC deformability was represented by the SS1/2:EImax ratio (Figure 1A–C). Analysis of the entire study cohort (Figure 1A; Total) revealed higher SS1/2:EImax values in the COVID-19 T0 group than in the Control group (*p* < 0.01), which represents lower deformability. The values of COVID-19 at T1 were significantly lower compared to T0 (*p* < 0.01) and comparable to the Control values. A sex-separated analysis of the study data revealed a similar pattern for the male cohort as for the Total sample (Figure 1B). The values of the female group showed no significant differences between Control and COVID-19 at T0 (*p* = 0.07), nor between COVID-19 at T0 vs. T1 (Figure 1C). Correlation analysis revealed a moderately negative correlation between SS1/2:EImax ratio and MCV (Pearson r: −0.3064).

### 3.3. Red Blood Cell Osmoscan

**Figure 2:**
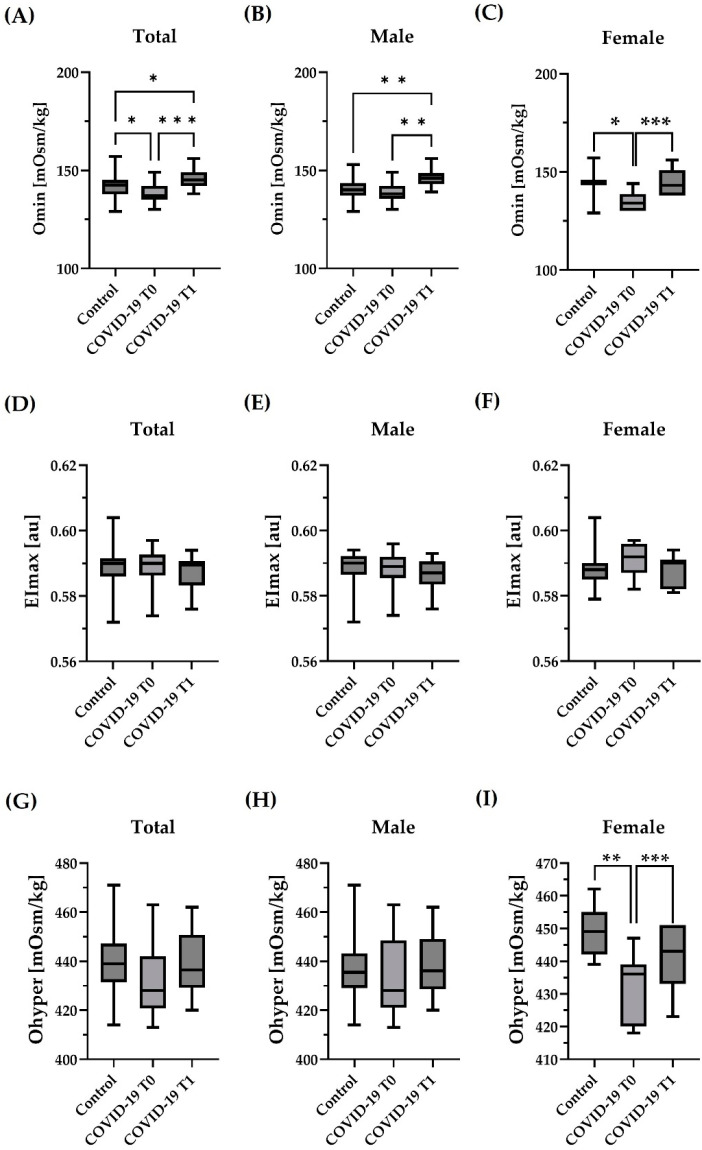
Influence of COVID-19 on deformability under an osmotic gradient (Osmoscan). Omin of (**A**) the total sample size showed lower values for COVID-19 at T0 compared to control and higher values in COVID-19 at T1 compared to T0 and control, respectively. (**B**) Males showed higher values for COVID-19 at T1 compared to T0 and control, respectively. (**C**) Females showed lower values for COVID-19 at T1 compared to control and higher values in COVID-19 at T1 compared to control. EImax of (**D**), the total sample size, I, the male cohort, and (**F**), the female cohort, showed no differences between the groups or timepoints. Ohyper of (**G**) the total sample size (**H**) the male cohort showed no differences between control and COVID-19 and no difference between the time points. (**I**) The values of the female cohort were significantly lower in COVID-19 at T0 compared to control and significantly higher in COVID-19 at T1 compared to T0. Sample size: (**A**,**D**,**G**): *n* = 42 control and *n* = 20 COVID-19; male cohort (**B**,**E**,**H**): *n* = 30 control, *n* = 13 COVID-19 T0 and *n* = 13 COVID-19 T1; female cohort (**C**,**F**,**I**): *n* = 12 control, *n* = 7 COVID-19 T0 and *n* = 7 COVID-19 T1. * *p* < 0.05; ** *p* < 0.01; *** *p* < 0.001.

Considering the total sample size, Omin was significantly lower in COVID-19 at T0 compared to Control (*p* < 0.05). At T1, values of COVID-19 were significantly higher compared to COVID-19 T0 (*p* < 0.001) but also compared to Control (*p* < 0.05) (Figure 2A). When only the male subjects were considered, Omin was higher in COVID-19 T1 compared to Control (*p* < 0.01) and higher compared to COVID-19 T0 (*p* < 0.01). In the female group, Omin was lower in COVID-19 at T0 compared to Control (*p* < 0.05). At T1, values were higher compared to T0 in COVID-19 (*p* < 0.001) (Figure 2C). The values of EImax obtained during osmoscan were not altered in COVID-19 compared to Control and the values of COVID-19 T0 were comparable to T1. No sex effects were detected (Figure 2D–F). Ohyper was comparable between Control and COVID-19 T0 and T1, respectively. This was observed for the Total sample size and for male participants, respectively (Figure 2G,H). In the female study cohort, Ohyper was significantly lower in COVID-19 at T0 compared to Control (*p* < 0.01). Values of female COVID-19 increased at T1 (*p* < 0.001) (Figure 2I).

### 3.4. Red Blood Cell Aggregation

**Figure 3:**
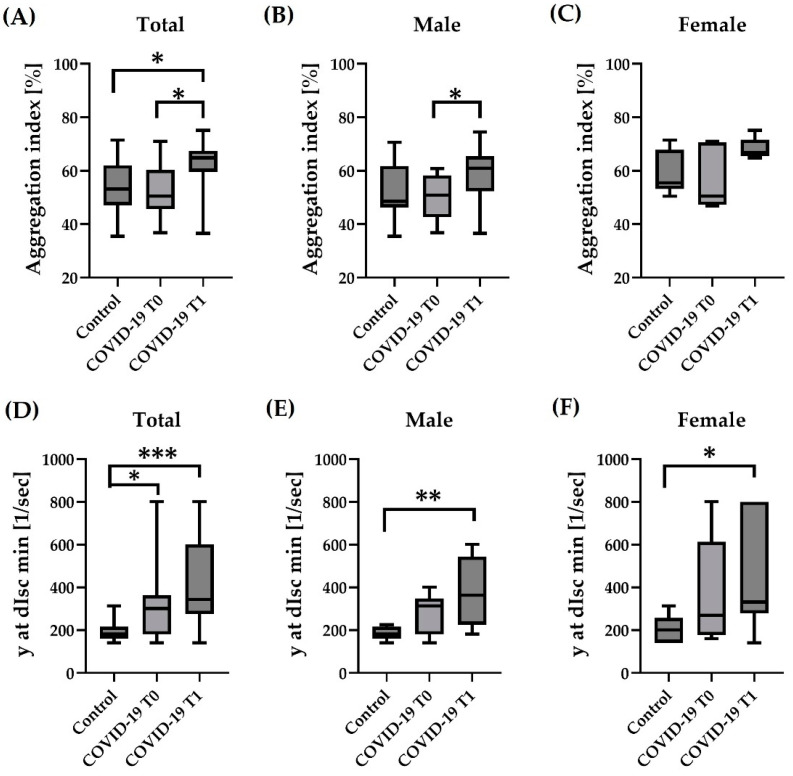
Influence of COVID-19 on RBC aggregation parameters. Aggregation index of (**A**) the total sample size was significantly higher in COVID-19 at T1 compared to T0 and control, respectively. (**B**) In the male cohort, the aggregation index was higher in COVID-19 at T1 compared to T0. (**C**) In the female cohort, the aggregation index did not differ between the groups or timepoints. γ at dIsc min of (**D**), the total sample was significantly higher in COVID-19 at T0 and T1 compared to Control. (**E**) Values of the male cohort were significantly higher in COVID-19 at T1 compared to Control, and (**F**) in the female cohort, values of COVID-19 at T1 were higher compared to Control. Sample size: (**A**,**D**): *n* = 24 control and *n* = 14 COVID-19; male cohort (**B**,**E**): *n* = 15 control, *n* = 8 COVID-19 T0 and *n* = 8 COVID-19 T1; female cohort (**C**,**F**): *n* = 9 control, *n* = 6 COVID-19 T0 and *n* = 6 COVID-19 T1. * *p* < 0.05; ** *p* < 0.01; *** *p* < 0.001.

Analysis of the Total study values showed that RBC aggregation index (AI%) was significantly higher in COVID-19 at T1 compared to T0 (*p* < 0.05) and compared to Control (*p* < 0.05), respectively (Figure 3A). Separate analyses of the sexes revealed higher values at T1 in male COVID-19 compared to T0 (*p* < 0.05) (Figure 3B). In female subjects, no difference in aggregation index was observed (Figure 3C). γ at dIsc min of Total sample size was higher in the COVID-19 group at T0 (*p* < 0.05) and T1 (*p* < 0.001) compared to Control, respectively (Figure 3D). γ at dIsc min of the male participants was significantly higher at T1 compared to Control (*p* < 0.01) (Figure 3E). γ at dIsc min of the female study group was significantly higher in COVID-19 at T1 compared to Controls (*p* < 0.05) (Figure 3F).

## 4. Discussion

SARS-CoV-2 infection and subsequent COVID-19 were associated with RBC morphology changes and impaired cell function that might result in reduced oxygen supply in the microcirculation [6]. To the best of our knowledge, follow-up investigations on hematological and hemorheological alterations have not been conducted after a mild disease course. However, as more and more studies show that COVID-19-related infections can persist over a long period of time, resulting in Long-COVID, this knowledge would add valuable information to further understand this phenomenon.

The results of the present study confirm alterations in hematological parameters, mainly related to cell volume and hemoglobin concentration, but also reduced deformability and increased aggregation after COVID-19 infection [5,6,9,10,20]. However, the findings also revealed that some changes persist for more than four months after infection, which might indicate long-term modifications of the RBC by the virus. The results indicate lower hemoglobin-related parameters and a reduced RBC volume in the COVID groups compared to control subjects, thus supporting recent data [5,21]. Interestingly, the data from this recent study also indicate that MCV increases 4 months after infection while hemoglobin parameters further decrease. SARS-CoV-2 infection of RBC is induced through the binding of the S1 spike protein of the virus and the Band-3 protein of the RBC membrane [22] and is able to interact with protoporphyrin IX. This binding possibly causes hemoglobin denaturation, resulting in a decreasing percentage of fully functional hemoglobin in oxygen transport [23]. In addition, an increased oxygen affinity of hemoglobin has been suggested as a compensatory mechanism for weakened lung function to ensure oxygen transport in COVID-19 patients [4]. This would be advantageous for loading the hemoglobin with oxygen in the lungs; however, would lead to a tighter binding of oxygen to the hemoglobin when it should be unloaded at the target cells [24]. In contrast to other studies [7], that investigated severe COVID-19 courses, no changes in RDW could be observed in this study. This supports the findings of other studies that RDW is related to disease severity [25]. These observed alterations in specific hematological parameters might be related to impaired oxygen transport and could partly explain various long-term symptoms such as loss of lung function and cardiorespiratory capacity, because RBC remain in the body due to their life span of about 120 days [20]. 

RBC deformability represents a unique cell characteristic allowing the passage of microcirculatory vessels with smaller diameters than the RBC [26]. It is described that proper deformability also determines the life span of the RBC itself but also affects blood circulation [27] since rigid cells might block small blood vessels [28]. Further, it is speculated whether RBC deformability affects oxygen supply within the microcirculation [29]. RBC deformability has been reported to be reduced after SARS-CoV-2 infection in critically ill patients but also in patients showing a rather mild disease course [5,6,9,20,30]. The results of the present investigation support recent findings showing reduced RBC deformability at T0 in COVID-19 patients compared to Control; but further, the data indicate that values tend to adjust to Control levels 4 months after the SARS-CoV-2 infection. Yet, RBC with only minor impairments of deformability may pass through the spleen without being noticed and stay in the blood circulation for months because of the long RBC life span. The altered physical properties of those cells might induce mechanical stress and affect the function of the spleen to filter out abnormal RBC. This might contribute to the long-term symptoms experienced by many COVID-19 patients [20]. Related to deformability measurements under an osmotic gradient (osmoscan), previous studies revealed a shift of the osmoscan curve to the left and downwards, even after a mild disease course, which might reflect stiffened cells [6]. The present results in part confirm these findings but suggest that these changes are reversible in the long term.

RBC deformability has been correlated to MCV [31] which is in part supported by the findings of the present study. Correlation analysis revealed a moderately negative correlation between SS1/2:EImax ratio and MCV, indicating a higher SS1/2:EImax ratio (lower deformability) might be associated with a low MCV. Indeed, in this study, MCV values increased over time, which was also observed for deformability parameters. However, it should be noted that other parameters related to deformability, including MCH and MCHC [31] were still reduced after 4 months, suggesting that the increase in RBC deformability during follow-up investigation is not exclusively related to improvements in hematological parameters. Previous studies described morphological changes of the RBC after SARS-CoV-2 infection [6,32,33] which might affect RBC deformability. It needs to be investigated whether these morphological changes are less prominent months after recovery because the affected RBC have meanwhile been removed from the circulation by regular erythrophagocytosis at the end of the RBC lifespan [34] or whether they persist in the long term. 

RBC aggregation is another parameter known to impact blood flow properties [35] and increased RBC aggregation has been reported for COVID-19 patients during the acute phase of the disease and is associated with hyperviscosity observed in these patients [6,9,10]. Aggregation parameters measured in the recent study add new information to the previous reports. While the former study of moderate disease courses after SARS-CoV-2 indicates unaltered aggregation indices soon after the infection [6], the recent data show an increase in aggregation index and a further increase in shear rate needed to separate formed aggregates four months after the first data acquisition. Higher aggregation in COVID-19 patients has been associated to an elevated fibrinogen level in COVID-19 patients [36]. However, it remains to be investigated whether fibrinogen levels or other factors, such as morphological alterations, are responsible for the present observations. The high individual range of γ at dIsc min might indicate that additional factors might affect the outcome. Among those, physical exercise, although there is no consensus in the literature [37,38], might be one parameter that might positively affect RBC aggregation [39]. The resumption or increase in physical activity after the COVID-19 disease may also explain the improvement in deformability between T0 and T1.

The presented data were further separated by sex to also address the known hematological and rheological differences between males and females [40,41]. These differences are associated with higher testosterone levels in males [42] and menstrual blood losses in females [43]; leading to a younger circulating RBC population and thus, higher deformability [40]. In total, differences between healthy Controls and COVID-19 subjects were more pronounced in the male cohort of this study than the female cohort. Especially certain hematological and deformability parameters were less different between female COVID-19 and respective control than male COVID-19 and respective control. The normal range for hemoglobin concentration in males is 13.8–17.2 g/dL. At T0, four males and at T1, eight males were below this range. The lowest value was 10.8 g/dL. However, there were also two controls below this range. In females, the normal range for hemoglobin concentration is 12.1–15.1 g/dL. Only one control was below this value but no COVID-19 female. This might lead to the assumption that men are more affected by the SARS-CoV-2 infection compared to women. And indeed, the disease severity and mortality rates seem to be higher in older males, while children, adolescents, and females tend to be mildly symptomatic or asymptomatic [11,44]. A sex-separated analysis of study data is thus advisable in order to evaluate physiological changes related to COVID-19 disease.

The results might be limited by the small number of female participants in this study. In addition, a comparative measurement four months later would have been useful for the controls as well, in order to exclude natural fluctuations. However, previous studies have shown a constancy of hematological values over a long period of time [40].

## 5. Conclusions

To the best of our knowledge, these are the first data to show altered hematological and rheological parameters after a mild COVID-19 infection that persist several months after infection. The changes include enduring reduced hb, MCH, and MCHC and higher RBC aggregation values that might possibly affect oxygen transport through the body. However, RBC deformability and MCV, which were significantly reduced shortly after the infection, seemed to recover in the months afterwards. The presented changes were more pronounced in male COVID-19 patients, suggesting that the infection has a higher impact on males than on females. Further investigations on the exact underlying mechanisms of prolonged altered RBC properties and sex differences are needed to develop specific therapies, especially considering the increasing number of patients suffering from Long-COVID.

## Figures and Tables

**Table 1:** Blood parameters of total participants. Mean values (SD) of *n* = 42 Control and *n* = 22 COVID-19.

Parameter	Total Control	Total COVID-19 T0	Total COVID-19 T1
RBC [×10^6^/µL]	4.73 (0.50)	4.69 (0.32)	4.72 (0.53)
Hb [g/dL]	14.55 (1.48)	14.11 (1.26)	13.41 (1.36) *^,†^
Hct [%]	42.78 (4.21)	41.01 (2.57)	41.70 (4.40)
MCV [fL]	90.61 (3.54)	87.45 (3.74) ##	88.39 (3.39) **^,†^
MCH [pg]	30.84 (1.54)	30.08 (1.99)	29.56 (2.79) **^,††^
MCHC [g/dL]	34.05 (1.59)	34.39 (1.45)	31.88 (3.02) **^,†^
RDW [%]	12.75 (0.68)	12.62 (0.58)	12.80 (0.75)

* *p* < 0.05; ** *p* < 0.01 COVID-19 T0 vs. T1, ## *p* < 0.01 Control vs. COVID-19 T0, ^†^ *p* < 0.05; ^††^ *p* < 0.01 Control vs. COVID-19 T1.

**Table 2:** Blood parameters of the male cohort. Mean values (SD). N = 30 Control; *n* = 15 COVID-19.

Parameter	Male Control	Male COVID-19 T0	Male COVID-19 T1
RBC [×10^6^/µL]	4.84 (0.51)	4.79 (0.31)	4.83 (0.57)
Hb [g/dL]	15.01 (1.42)	14.38 (1.23)	13.56 (1.38) *^,††^
Hct [%]	43.69 (4.22)	41.57 (2.04)	42.52 (4.64)
MCV [fL]	90.45 (3.51)	87.01 (3.78) ##	88.10 (3.31) *
MCH [pg]	31.09 (1.54)	30.07 (2.11)	28.27 (3.03) *^,†††^
MCHC [g/dL]	34.39 (1.63)	34.55 (1.60)	31.47 (3.49) *^,††^
RDW [%]	12.71 (0.66)	12.63 (0.66)	12.91 (0.87)

* *p* < 0.05 COVID-19 T0 vs. T1, ## *p* < 0.01 Control vs. COVID-19 T0, ^††^ *p* < 0.01; ^†††^ *p* < 0.001 Control vs. COVID-19 T1.

**Table 3:** Blood parameters in female participants with data representing Mean (SD). *n* = 12 Control; *n* = 7 COVID-19.

Parameter	Female Control	Female COVID-19 T0	Female COVID-19 T1
RBC [×10^6^/µL]	4.45 (0.38)	4.50 (0.26)	4.49 (0.37)
Hb [g/dL]	13.42 (0.94)	13.54 (1.20)	13.10 (1.35)
Hct [%]	40.49 (3.33)	39.80 (3.29)	39.96 (3.50)
MCV [fL]	91.03 (3.73)	88.40 (3.76)	89.00 (3.73) *
MCH [pg]	30.21 (1.42)	30.09 (1.87)	29.19 (2.30)
MCHC [g/dL]	33.18 (1.10)	34.03 (1.08)	32.76 (1.51)
RDW [%]	12.84 (0.74)	12.60 (0.42)	12.56 (0.32)

* *p* < 0.05 COVID-19 T0 vs. T1.

## Data Availability

The data that support the findings of this study are available upon reasonable request from the corresponding author. The data are not publicly available due to privacy or ethical restrictions.
